# Reconsidering low-dose aspirin therapy for cardiovascular disease: a study protocol for physician and patient behavioral change

**DOI:** 10.1186/1748-5908-6-65

**Published:** 2011-06-26

**Authors:** Brittany Folks, William G LeBlanc, Elizabeth W Staton, Wilson D Pace

**Affiliations:** 1 University of Colorado Denver, Department of Family Medicine, 12681 East 17th Ave. Bldg A01, Mail Stop 496, Aurora, CO 80045-0508, USA

## Abstract

**Background:**

There are often disparities between current evidence and current practice. Decreasing the gap between desired practice outcomes and observed practice outcomes in the healthcare system is not always easy. Stopping previously recommended or variably recommended interventions may be even harder to achieve than increasing the use of a desired but under-performed activity. For over a decade, aspirin has been prescribed for primary prevention of cardiovascular disease and for patients with the coronary artery disease risk equivalents; yet, there is no substantial evidence of an appropriate risk-benefit ratio to support this practice. This paper describes the protocol of a randomized trial being conducted in six primary care practices in the Denver metropolitan area to examine the effectiveness of three interventional strategies to change physician behavior regarding prescription of low-dose aspirin.

**Methods:**

All practices received academic detailing, one arm received clinician reminders to reconsider aspirin, a second arm received both clinician and patient messages to reconsider aspirin. The intervention will run for 15 to 18 months. Data collected at baseline and for outcomes from an electronic health record will be used to assess pre- and post-interventional prescribing, as well as to explore any inappropriate decrease in aspirin use by patients with known cardiovascular disease.

**Discussion:**

This study was designed to investigate effective methods of changing physician behavior to decrease the use of aspirin for primary cardiovascular disease prevention. The results of this study will contribute to the small pool of knowledge currently available on the topic of ceasing previously supported practices.

**Trial Registration:**

ClinicalTrials.gov: NCT01247454

## Background

Cardiovascular disease is the leading cause of death for men and women in the United States. In 2002, the United States Preventive Services Task Force (USPSTF) began recommending low-dose aspirin as a primary prevention measure in patients at high risk of developing coronary artery disease [1]. However, there are many well-designed studies that do not support the use of aspirin for primary prevention of cardiovascular events in patients with no known coronary artery disease [2-6], including patients with conditions considered to confer a risk equivalent to coronary artery disease, such as diabetes [7-9], peripheral artery disease [7], and chronic kidney disease [10]. Meta-analyses of many studies have also shown inconsistent results [11-13]. In addition to the uncertainty of efficacy, research has shown that the benefits of low-dose aspirin for primary prevention may not appropriately outweigh the harms [14]. The Food and Drug Administration (FDA) has denied requests to approve aspirin for the primary prevention of cardiovascular events twice, once in 1998 and again in 2003, due to lack of evidence supporting its efficacy [15,16]. Since 2003, no new large studies that support the use of low-dose aspirin therapy have been published, though several that do not demonstrate a protective effect of low-dose aspirin have been completed [4,7,8,10]. However, aspirin has continued to be recommended for this use by the American Heart Association [17], the USPSTF [18], the American Stroke Association [17], and the American Diabetes Association [19], and is prescribed for primary prevention by physicians worldwide.

There is often a disparity between current evidence and current practice, but making changes to the system can prove difficult. Research has revealed that physicians' behavior is relatively resistant to change due to a variety of internal and external factors [20-24]. However, some interventional methods have been more efficacious than others. Mere passive dissemination of information is typically ineffective, whereas active interventions have been shown to be more successful [25-27]. While many physicians believe that clinical guidelines are beneficial to their practice and patient outcomes [28,29], clinical guidelines alone do not seem to impact physician behavior [30]. Furthermore, guidelines that recommend the elimination of old behavior may be more difficult to implement than guidelines that recommend the addition of a new behavior [21].

Sittig, *et al. *conducted a study to test clinical decision support systems on reducing the use of inappropriate medications in various groups of patients [24]. The study added to the evidence that clinical decision support systems are effective [25,31,32]. Reminders that utilize similar electronic systems [27,30,31,33-35] and educational outreach (*i.e.*, academic detailing) [25-27,30,34,36] have produced significant results. Interventions that are patient mediated have been successful as well [25]. Multi-faceted techniques involving two or more interventions have also proven more effective than single interventions [25-27,30,34,36].

Until recently, all primary care physicians within the University of Colorado Hospital ambulatory system received reminders through a clinical decision support system to consider aspirin therapy for all patients with diabetes mellitus, peripheral artery disease, chronic kidney disease, or a calculated Framingham Risk Score >20%. Based on the lack of evidence supporting this practice, the group of Family Medicine and General Internal Medicine physicians overseeing this system decided to remove the reminder message from the electronic medical record. No active, systematic approach to stop this previously recommended behavior has been undertaken. Members of the Clinical Decision Support group indicated that they believe simply discontinuing the recommendation would have no impact on clinical care, and that the use of low-dose aspirin for primary prevention would otherwise continue. Therefore, this study was designed to actively disseminate to clinicians the new University of Colorado Hospital primary care recommendations regarding the use of aspirin for primary prevention.

The aims of this project are:

1. Assess the current use of aspirin therapy for primary and secondary cardiovascular disease prevention in six General Internal Medicine and Family Medicine clinics within the University of Colorado Hospital system using electronic health record data;

2. Develop messages concerning the appropriate use of aspirin for cardiovascular disease prevention for academic detailing to clinicians, a point-of-care decision support aid for clinicians, and a patient activation form; and

3. Test the effectiveness of these interventions to improve the evidence based use of aspirin for primary and secondary prevention of cardiovascular disease using a three arm randomized trial.

The three intervention arms will be as follows:

1. Academic detailing only;

2. Academic detailing plus a message to the physician in the clinical decision support system asking the clinician to reconsider the aspirin therapy; and

3. Academic detailing, plus the physician message, plus a patient activation form that includes a message to the patient to ask his or her physician about use of aspirin for primary prevention.

The academic detailing presentation (http://www.dartnet.info/media/LowDoseASAandCVDpreventionSlides.pdf) and one page information sheet (http://www.dartnet.info/media/LowDoseASAEvidenceSummaryforClinicians.pdf) are available online.

The null hypotheses for the project, stated as null hypotheses are:

1. There will be no significantly different decrease in the use of low-dose aspirin therapy for primary prevention across the three intervention arms from baseline to completion of the project;

2. There will be no significant differences in the percentage of patients with diabetes mellitus over age 44 years on low-dose aspirin therapy for primary prevention across the three arms of the study, as shown by repeated measures from baseline to completion of the project; and

3. There will be no significant difference in the percentage of patients with known ischemic heart disease treated with low-dose aspirin between the three arms of the study at baseline and at the end of the study (*i.e.*, there will be no erosion in the appropriate use of aspirin due to the intervention to decrease aspirin use for primary prevention).

## Design and methods

### Design

This project is primarily designed as comparative effectiveness trial with all intervention sites receiving education concerning a system wide decision to stop recommending aspirin for primary prevention of cardiovascular disease. Six University of Colorado primary care practices in the Denver metro area were block randomized by baseline number of patients on aspirin for primary prevention into one of three intervention arms:

1. Academic detailing and cessation of the primary prevention reminder within the point-of-care clinical decision support system only;

2. Academic detailing with a message asking clinicians to consider stopping aspirin therapy for primary prevention embedded in the point-of-care clinical decision support system; or

3. Academic detailing with the point-of-care message for clinicians as described in arm two above, and a short patient activation form to be given to patients prior to a visit, which asks them to check with their provider concerning their use of aspirin for primary prevention.

The academic detailing consisted of a presentation to the clinicians and staff of each office by the study principal investigator and co-investigator (for larger practices more than one presentation was made), as well as the development and distribution to all primary care clinicians of a one-page information sheet concerning the lack of evidence for low-dose aspirin for primary prevention of cardiovascular disease (CVD). The patient activation form, shown in Additional File 1, was requested by one of the primary care practices prior to this study. All messages on the form, including the aspirin message, were reviewed by clinicians from all primary care practices. Practices were free to request that the forms be turned off at any time during the study, but none did so.

One primary care practice in the system elected to not receive the academic detailing or to participate in the project. This practice will be utilized as a non-randomized temporal control for the intervention practices. An interim analysis will be conducted at six months and the final analysis will be conducted at 15 to 18 months.

**Figure 1:**
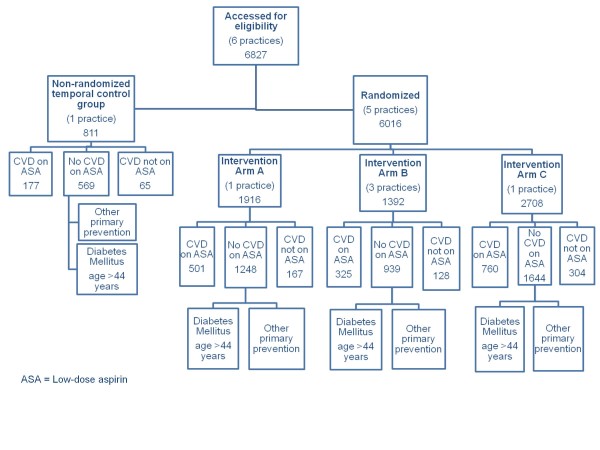
**CONSORT diagram of the study**.

All practices involved in the study were divided into three groups to match the number of patients at baseline on aspirin for primary prevention as closely as possible. After the academic detailing was completed at all sites, practice names were placed on identical cards and blindly drawn from a box by a study team member to allocate them to the patient activation, clinician-only reminder, and academic detailing alone groups. The consort diagram of number of patients randomized to each intervention is shown in Figure 1.

### Participants

Pre-intervention data were collected through the electronic medical record system (N = 6,827). Patients were separated into categories based on two criteria as listed in their charts: a once daily dosing of low-dose aspirin and a diagnosis of CVD based on appropriate International Classification of Disease, Ninth Revision (ICD-9) codes (both coronary artery disease and thrombotic cerebrovascular disease). Persons who met both criteria were classified as 'CVD and on aspirin' (N = 1,763). Persons who met the criteria of low-dose aspirin but had no associated cardiovascular diagnosis were classified as 'No CVD but on aspirin' (N = 4,400). Those who had no documentation of aspirin or other anti-platelet therapy, but who carried a cardiovascular diagnosis were classified as 'CVD but not on aspirin' (N = 664). The study is targeted to change care in the 'No CVD but on aspirin' group specifically, but will observe the 'CVD and on aspirin' and 'CVD but not on aspirin' groups to monitor for any change, particularly an unwanted drop in use of anti-platelet aspirin therapy in patients with known CVD.

For the outcome variable of change to recommended aspirin therapy, a sample size of 800 per arm provides 80% power to detect a 7% difference between any two groups. We believe that any change under 10% is not clinically significant; therefore, the ability to show an even smaller statistically significant change indicates that this study has enough power to detect meaningful change in aspirin therapy.

### Data collection

Our pre-intervention data were collected using the University of Colorado Hospital's Allscripts database and the QED Clinical, Inc. (DBA-CINA) Clinical Data Repository maintained by the Department of Family Medicine. Our post-intervention data will be collected using the same system. The intervention will run for 15 to 18 months.

### Data analysis approach

The primary end point of this project is the number (and percent) of individuals currently on aspirin therapy for primary CVD prevention that stop this therapy and do not receive a new CVD diagnosis. All practices in this project perform regular medication reconciliation at virtually all visits; thus, aspirin therapy is regularly recorded in the patient drug list. Electronic health record (EHR) data can reasonably identify patients on aspirin as well as identify those individuals with an appropriate diagnosis for this therapy. Thus, we will conduct our analyses at the patient level. Primary analysis will be based on the 'No CVD on aspirin' cohorts by intervention arm. Secondary analyses will include a comparison to the temporal control and sub-group analysis comparing individuals with diabetes mellitus, peripheral vascular disease and chronic kidney disease versus all others in the 'No CVD on aspirin' cohort. Data are available to calculate a Framingham risk score [37] for essentially all participants without a 'CVD equivalent' diagnosis. If there is a differential effect between the CVD equivalent and all others in the 'No CVD on aspirin' cohort, a sub-analysis that includes the Framingham risk score will be undertaken. An interim analysis conducted at six months did not demonstrate any decrease in aspirin usage among patients with a CVD diagnosis, a predetermined early stopping point.

The primary analysis will include only patients identified as being on aspirin prior to 1 April 2009, when the decision support aspirin message was changed. Aspirin therapy status will be evaluated at six and twelve months after the intervention is initiated at each site. The outcome variable for the analysis will be presence/absence of aspirin therapy at final observation time. We will use a logistic regression approach to determine whether likelihood of aspirin cessation differs among treatment groups, adjusting for clinic (fixed effect), and patient clinical and sociodemographic covariates (age, gender). Time from baseline to last observation will be included as a covariate in the primary analysis. Patients who do not have a follow-up visit during this period will initially be excluded from the analysis, but a sensitivity analysis will be performed, assuming patients with no follow-up visit are still on aspirin therapy. Clinic will initially be included as a fixed effect in analyses because the total number of clinics is too few to achieve stable estimates of covariance components for multilevel modeling. However, additional analyses will be carried out using multilevel modeling with patients nested within physicians because there are an adequate number to include physician as a random effect. Additionally, we will explore using a Cox proportional hazards model with time to cessation as the outcome.

### Human subjects protection

This study was approved by the Colorado Multiple Institution Review Board. It was also registered on ClinicalTrials.gov, identifier: NCT01247454. The study was granted a waiver of consent at the patient level.

## Discussion

One of the six practices withdrew from the study prior to the randomization and implementation of the interventions, reducing the patient size (N = 6,016). For this practice, the primary prevention reminder was stopped within the point-of-care clinical decision support system. This practice was treated as a non-randomized temporal control for the intervention practices. Even without randomizing this practice, the power of the study will remain sufficient.

Much research has been done regarding physician behavior change and implementation of new techniques, treatment, and therapies. However, there is not a great deal of literature surrounding effective methods for discontinuation of current practices. Effecting a change in aspirin usage in one or more groups will result in a better understanding of the approaches and efforts needed to stop the use of a previously recommended therapy. If a change in aspirin usage is not detected, a follow-up qualitative analysis at the provider level may provide a better understanding of why providers and patients were unwilling to stop a potentially harmful treatment.

## Competing interests

The authors declare that they have no competing interests.

## Authors' contributions

BF conceived the study, conducted the academic detailing, and drafted the manuscript. BL planned, described, and will conduct the statistical analysis. ES participated in the design of the patient activation form and helped to draft the manuscript. WP participated in the design of the study and the patient activation form and helped to draft the manuscript. All authors read and approved the final manuscript.

## Supplementary Material

Additional file 1 The patient activation form Click here for file

## References

[B1] Hayden M Pignone M Phillips C Mulrow C Aspirin for the primary prevention of cardiovascular events: a summary of the evidence for the U.S. Preventive Services Task Force Ann Intern Med 2002 136 161 172 11790072 10.7326/0003-4819-136-2-200201150-00016

[B2] Hansson L Zanchetti A Carruthers SG Dahlof B Elmfeldt D Julius S Menard J Rahn KH Wedel H Westerling S Effects of intensive blood-pressure lowering and low-dose aspirin in patients with hypertension: Principal results of the Hypertension Optimal Treatment (HOT) randomised trial. HOT Study Group Lancet 1998 351 1755 1762 10.1016/S0140-6736(98)04311-6 9635947

[B3] Peto R Gray R Collins R Wheatley K Hennekens C Jamrozik K Warlow C Hafner B Thompson E Norton S Randomised trial of prophylactic daily aspirin in British male doctors Br Med J (Clin Res Ed) 1988 296 313 316 10.1136/bmj.296.6618.313 PMC2544821 3125882

[B4] Ridker PM Cook NR Lee IM Gordon D Gaziano JM Manson JE Hennekens CH Buring JE A randomized trial of low-dose aspirin in the primary prevention of cardiovascular disease in women N Engl J Med 2005 352 1293 1304 10.1056/NEJMoa050613 15753114

[B5] Final report on the aspirin component of the ongoing Physicians' Health Study. Steering Committee of the Physicians' Health Study Research Group N Engl J Med 1989 321 129 135 2664509 10.1056/NEJM198907203210301

[B6] Anonymous Thrombosis prevention trial: randomised trial of low-intensity oral anticoagulation with warfarin and low-dose aspirin in the primary prevention of ischaemic heart disease in men at increased risk. The Medical Research Council's General Practice Research Framework.[see comment] Lancet 1998 351 233 241 9457092

[B7] Belch J MacCuish A Campbell I Cobbe S Taylor R Prescott R Lee R Bancroft J MacEwan S Shepherd J Macfarlane P Morris A Jung R Kelly C Connacher A Peden N Jamieson A Matthews D Leese G McKnight J O'Brien I Semple C Petrie J Gordon D Pringle S MacWalter R The prevention of progression of arterial disease and diabetes (POPADAD) trial: factorial randomised placebo controlled trial of aspirin and antioxidants in patients with diabetes and asymptomatic peripheral arterial disease BMJ 2008 337 a1840 10.1136/bmj.a1840 18927173 PMC2658865

[B8] Ogawa H Nakayama M Morimoto T Uemura S Kanauchi M Doi N Jinnouchi H Sugiyama S Saito Y Low-dose aspirin for primary prevention of atherosclerotic events in patients with type 2 diabetes: a randomized controlled trial JAMA 2008 300 2134 2141 10.1001/jama.2008.623 18997198

[B9] Sacco M Pellegrini F Roncaglioni MC Avanzini F Tognoni G Nicolucci A Primary prevention of cardiovascular events with low-dose aspirin and vitamin E in type 2 diabetic patients: results of the Primary Prevention Project (PPP) trial Diabetes Care 2003 26 3264 3272 10.2337/diacare.26.12.3264 14633812

[B10] Ethier J Bragg-Gresham JL Piera L Akizawa T Asano Y Mason N Gillespie BW Young EW Aspirin prescription and outcomes in hemodialysis patients: the Dialysis Outcomes and Practice Patterns Study (DOPPS).[see comment] American Journal of Kidney Diseases 2007 50 602 611 10.1053/j.ajkd.2007.07.007 17900460

[B11] Baigent C Blackwell L Collins R Emberson J Godwin J Peto R Buring J Hennekens C Kearney P Meade T Patrono C Roncaglioni MC Zanchetti A Aspirin in the primary and secondary prevention of vascular disease: collaborative meta-analysis of individual participant data from randomised trials Lancet 2009 373 1849 1860 10.1016/S0140-6736(09)60503-1 19482214 PMC2715005

[B12] Bartolucci AA Howard G Meta-analysis of data from the six primary prevention trials of cardiovascular events using aspirin Am J Cardiol 2006 98 746 750 10.1016/j.amjcard.2006.04.012 16950176

[B13] Berger JS Roncaglioni MC Avanzini F Pangrazzi I Tognoni G Brown DL Aspirin for the primary prevention of cardiovascular events in women and men: a sex-specific meta-analysis of randomized controlled trials JAMA 2006 295 306 313 10.1001/jama.295.3.306 16418466

[B14] Patrono C Garcia Rodriguez LA Landolfi R Baigent C Low-dose aspirin for the prevention of atherothrombosis N Engl J Med 2005 353 2373 2383 10.1056/NEJMra052717 16319386

[B15] Fleming T Nissen SE Borer JS Armstrong PW Report from the 100th Cardiovascular and Renal Drugs Advisory Committee meeting: US Food and Drug Administration: December 8-9, 2003 Gaithersburg, MD Circulation 2004 109 e9004 9005 14734515 10.1161/01.CIR.0000118364.89571.16

[B16] Aspirin should it be used for primary prevention in diabetics? http://www.theheart.org/article/912299.do

[B17] Goldstein LB Adams R Alberts MJ Appel LJ Brass LM Bushnell CD Culebras A Degraba TJ Gorelick PB Guyton JR Hart RG Howard G Kelly-Hayes M Nixon JV Sacco RL Primary prevention of ischemic stroke: a guideline from the American Heart Association/American Stroke Association Stroke Council: cosponsored by the Atherosclerotic Peripheral Vascular Disease Interdisciplinary Working Group; Cardiovascular Nursing Council; Clinical Cardiology Council; Nutrition, Physical Activity, and Metabolism Council; and the Quality of Care and Outcomes Research Interdisciplinary Working Group: the American Academy of Neurology affirms the value of this guideline Stroke 2006 37 1583 1633 16675728 10.1161/01.STR.0000223048.70103.F1

[B18] Aspirin for the prevention of cardiovascular disease: U.S. Preventive Services Task Force recommendation statement Ann Intern Med 2009 150 396 404 19293072 10.7326/0003-4819-150-6-200903170-00008

[B19] Colwell JA Aspirin therapy in diabetes Diabetes Care 2003 26 Suppl 1 S87 88 12502626 10.2337/diacare.26.2007.s87

[B20] Berenholtz S Pronovost PJ Barriers to translating evidence into practice Curr Opin Crit Care 2003 9 321 325 10.1097/00075198-200308000-00012 12883289

[B21] Cabana MD Rand CS Powe NR Wu AW Wilson MH Abboud PA Rubin HR Why don't physicians follow clinical practice guidelines? A framework for improvement JAMA 1999 282 1458 1465 10.1001/jama.282.15.1458 10535437

[B22] Gronseth GS From evidence to action NeuroRx 2004 1 331 340 15717035 10.1602/neurorx.1.3.331 PMC534934

[B23] Poses RM One size does not fit all: questions to answer before intervening to change physician behavior Joint Commission Journal on Quality Improvement 1999 25 486 495 10481818 10.1016/s1070-3241(16)30463-1

[B24] Sittig DF Krall MA Dykstra RH Russell A Chin HL A survey of factors affecting clinician acceptance of clinical decision support BMC Med Inform Decis Mak 2006 6 6 10.1186/1472-6947-6-6 16451720 PMC1403751

[B25] Bero LA Grilli R Grimshaw JM Harvey E Oxman AD Thomson MA Getting research findings into practice: Closing the gap between research and practice: An overview of systematic reviews of interventions to promote the implementation of research findings BMJ 1998 317 465 468 9703533 10.1136/bmj.317.7156.465 PMC1113716

[B26] Bloom BS Effects of continuing medical education on improving physician clinical care and patient health: a review of systematic reviews Int J Technol Assess Health Care 2005 21 380 385 16110718 10.1017/s026646230505049x

[B27] Grimshaw JM Eccles MP Walker AE Thomas RE Changing physicians' behavior: what works and thoughts on getting more things to work J Contin Educ Health Prof 2002 22 237 243 10.1002/chp.1340220408 12613059

[B28] Newton J Knight D Woolhead G General practitioners and clinical guidelines: a survey of knowledge, use and beliefs Br J Gen Pract 1996 46 513 517 8917869 PMC1239745

[B29] Siriwardena AN Clinical guidelines in primary care: a survey of general practitioners' attitudes and behaviour Br J Gen Pract 1995 45 643 647 8745861 PMC1239465

[B30] Smith WR Evidence for the effectiveness of techniques To change physician behavior Chest 2000 118 8S 17S 10.1378/chest.118.2_suppl.8S 10939994

[B31] Elson RB Connelly DP Computerized patient records in primary care. Their role in mediating guideline-driven physician behavior change Arch Fam Med 1995 4 698 705 10.1001/archfami.4.8.698 7620600

[B32] Johnston ME Langton KB Haynes RB Mathieu A Effects of computer-based clinical decision support systems on clinician performance and patient outcome. A critical appraisal of research Ann Intern Med 1994 120 135 142 8256973 10.7326/0003-4819-120-2-199401150-00007

[B33] Austin SM Balas EA Mitchell JA Ewigman BG Effect of physician reminders on preventive care: meta-analysis of randomized clinical trials Proc Annu Symp Comput Appl Med Care 1994 121 124 PMC2247964 7949904

[B34] Davis DA Taylor-Vaisey A Translating guidelines into practice. A systematic review of theoretic concepts, practical experience and research evidence in the adoption of clinical practice guidelines CMAJ 1997 157 408 416 9275952 PMC1227916

[B35] Shea S DuMouchel W Bahamonde L A meta-analysis of 16 randomized controlled trials to evaluate computer-based clinical reminder systems for preventive care in the ambulatory setting Journal of the American Medical Informatics Association 1996 3 399 409 10.1136/jamia.1996.97084513 8930856 PMC116324

[B36] Oxman AD Thomson MA Davis DA Haynes RB No magic bullets: a systematic review of 102 trials of interventions to improve professional practice CMAJ 1995 153 1423 1431 7585368 PMC1487455

[B37] D'Agostino RB Sr Vasan RS Pencina MJ Wolf PA Cobain M Massaro JM Kannel WB General cardiovascular risk profile for use in primary care: the Framingham Heart Study Circulation 2008 117 743 753 10.1161/CIRCULATIONAHA.107.699579 18212285

