# Supplementary material for: Reconsidering low-dose aspirin therapy for cardiovascular disease: a study protocol for physician and patient behavioral change
Source: Implement Sci. 2011 Jun 26;6:65. doi: 10.1186/1748-5908-6-65 (PMC3141566; doi:10.1186/1748-5908-6-65)
Supplement: Additional file 1 — The patient activation form [file 1748-5908-6-65-S1.PDF]

The patient activation form shown below includes an area to reconcile medications prescribed with medications that the patient is actually taking. (The medication list includes immunizations ordered for the patient.) In addition, the form presents health messages based on information contained in the electronic health record, including the aspirin message. In this sample, the patient was prompted to ask his/her provider about the use of low-dose aspirin because he/she did not have a diagnosis of coronary artery disease or thrombotic cerebrovascular disease. The patient also received messages regarding his/her blood pressure, cholesterol level, and pneumonia vaccination record.

[illegible] **DOB :** [illegible]/[illegible]/[illegible] **MRN:** [illegible] [illegible]

Please cross out any medications not currently taking.

Medication Name	Dose	Frequency	Need refills	New dose	I don't Know
Aspirin	81 MG		☐	☐	☐
Gardasil		INJECT INTRAMUSCULARLY AS DIRECTED.	☐	☐	☐
Lansoprazole	30 MG	TAKE 1 CAPSULE TWICE DAILY	☐	☐	☐
Lantus	100 UNIT/ML	INJECT 10 UNIT *Daily	☐	☐	☐
Lisinopril	10 MG	TAKE 1 TABLET DAILY FOR BLOOD PRESSURE.	☐	☐	☐
MetFORMIN HCl	1000 MG	TAKE 1 TABLET EVERY 12 HOURS DAILY.	☐	☐	☐
OneTouch Ultra Test		USE 1 STRIP AS NEEDED	☐	☐	☐

Some good news:

1. Not smoking is very important for your health. Our records show that you do not smoke.
2. Our records show that your cholesterol is fine.

For your health:

1. New studies show that you may not need daily aspirin therapy. Please ask your provider about aspirin.
2. Our records show that your blood pressure is too high. Please talk with your provider about your blood pressure.
3. Our records show that your cholesterol is too high. Please talk with your provider about your cholesterol.
4. Some pneumonias can be prevented with a vaccine. Please ask your provider about a pneumonia shot.

Notes
